# Clival Osteomyelitis Secondary to Isolated Sphenoid Sinusitis Caused by Nocardia veterana in an Immunocompetent Patient: A Case Report

**DOI:** 10.7759/cureus.81352

**Published:** 2025-03-28

**Authors:** Vita L Dingerkus, Daman Bhatia, Raymond Kim, Vinod Khanijow, Brian Sloan

**Affiliations:** 1 Ophthalmology, University of Auckland, Auckland, NZL; 2 Ophthalmology, City Hospital Zurich, Zurich, CHE; 3 Otorhinolaryngology, University of Auckland, Auckland, NZL; 4 Otorhinolaryngology, Auckland District Health Board, Auckland, NZL; 5 Pathology, University of Auckland, Auckland, NZL; 6 Ophthalmology, Auckland District Health Board, Auckland, NZL

**Keywords:** abducens nerve palsy, clival osteomyelitis, isolated sphenoid sinusitis, nocardia, optic neuropathy

## Abstract

Nocardiosis is an infectious disease caused by filamentous, saprophytic bacteria that classically affects the lungs, central nervous system, and skin as an opportunistic infection in immunocompromised patients. Although it can affect any organ system in general, osteomyelitis is not commonly seen and even more rarely involves the skull.

We report the uncommon case of a clival osteomyelitis secondary to isolated sphenoid sinusitis caused by *Nocardia veterana* in an 85-year-old immunocompetent female who presented with right-sided facial pain, abducens nerve palsy, and eventually loss of vision.

## Introduction

Clival osteomyelitis, a rare skull base infection, is an uncommon but life-threatening complication of untreated malignant otitis externa or paranasal sinus infection. It is most commonly caused by *Pseudomonas aeruginosa* and *Staphylococcus spp*., but especially in immunocompromised patients, one must consider other less common pathogens, such as fungal microorganisms. It is rarely caused by *Nocardia species (spp.)*, which are filamentous, saprophytic, Gram-positive aerobic bacteria. *Nocardia* exists ubiquitously in the environment and classically affects the lungs or skin as an opportunistic infection in immunocompromised patients. Due to *Nocardia's* affinity to the central nervous system and the close anatomic relationship of the clivus and the sphenoid sinus to the optic nerve, orbit, and cavernous sinus, there is a high risk of secondary orbital manifestation [1]. We report the uncommon case of a clival osteomyelitis secondary to isolated sphenoid sinusitis caused by *Nocardia veterana* in an 85-year-old immunocompetent female who presented initially with facial pain and right abducens nerve palsy and ultimately right visual loss.

## Case presentation

An 85-year-old female patient presented initially to the emergency department (ED) at a peripheral secondary care hospital. She gave an approximately three-month history of worsening right facial and forehead pain that could no longer be adequately managed by the primary care physician and no other accompanying symptoms. She had chronic hyponatremia but was otherwise healthy. There was no history of malignancy or diabetes mellitus. Early differential diagnoses included giant cell arteritis (GCA), sinusitis, and trigeminal neuralgia, and as a result, she was initially seen by specialists in neurology, otolaryngology (ORL), and rheumatology.

**Figure 1:**
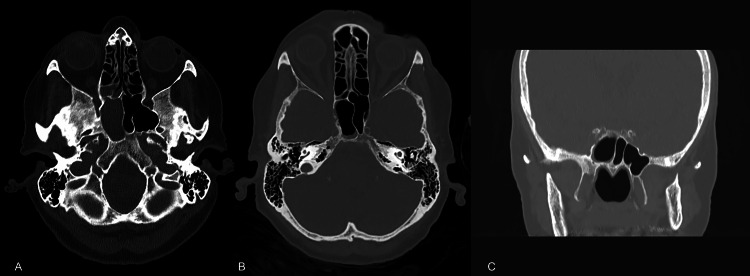
CT scan findings A: axial and coronal CT scans showing the greatest extent of right sphenoid sinusitis early after initial presentation; B: after antibiotic treatment two months later; B, C: bony erosion in the lateral sinus wall despite the finding of treated sinusitis.

Neurological clinical examination excluded trigeminal neuralgia. The C-reactive protein (CRP) was initially elevated (38 mg/l). A (plain) CT scan identified a right sphenoid sinusitis (Figure 1A). 

She was then treated for both GCA and sinusitis with prednisone and amoxicillin and had a temporal artery biopsy (TAB), which was negative for vasculitis. She initially felt better on prednisone at the initial dosage of 40 mg, but this improvement was not sustained. Slow tapering was recommended (5 mg per month) by the rheumatologist. The patient refused a contralateral TAB. Temporal artery ultrasound three weeks after presentation was normal, and the diagnosis of GCA was considered to be unlikely, so the prednisone was tapered to zero.

Surgical drainage of the sphenoid sinus was considered, but the follow-up CT scan of the sinuses two months after the initial presentation reported only a rim of fluid within the right sphenoid sinus, and the repeat endoscopic finding was normal. Accordingly, the isolated sphenoid sinusitis was considered adequately treated (Figure 1B). 

One month later (four months after the ED presentation), the patient presented again to the ORL clinic with new-onset diplopia and right abduction restriction. CT head and venograms revealed an erosion of the right superolateral sphenoid sinus wall, still with minimal mucosal edema present in the right sphenoid (Figure 1C). An MRI of the brain was performed as well to exclude pathology in the cavernous sinus or other causes for her symptoms. This was reported to be normal, although the technical quality of the MRI was not optimal due to motion artifacts.

Shortly after, she reported blurred vision in the right eye and ocular pain. Her right vision was mildly decreased compared to the left (uncorrected visual acuity of 6/12 versus 6/9) with normal anterior and posterior segment findings, but a right relative afferent pupillary defect (RAPD) and reduced green-red perception were noted, as well as a right abducens nerve palsy. A neuroophthalmological follow-up was planned; however, her vision decreased in the following two weeks to no perception of light (NPL). The CRP remained normal, and she was again treated as an atypical GCA with corticosteroids.

**Figure 2:**
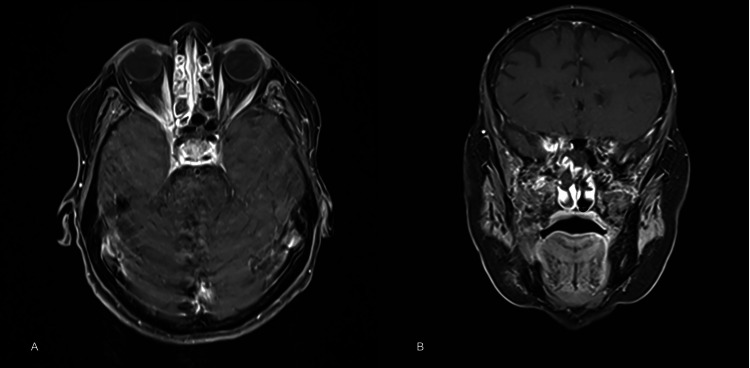
MRI findings A: MRI head and orbits revealing relatively diffuse soft tissue thickening and enhancement at right orbital apex in axial planes; B: coronal planes. The right optic nerve sheath is mildly enhancing compared to the left, with a normal appearance in signal and caliber of the optic nerve itself.

A further MRI (performed five months after ED presentation) identified subtle soft tissue thickening and enhancement of the right orbital apex involving the region of the rectus muscles, optic nerve sheath, cavernous sinus, and proximal right maxillary nerve, potentially explaining all her symptoms (Figure 2).

Repeated right TAB shortly afterward was negative, and the differential diagnosis of GCA was abandoned, and the diagnosis of Tolosa-Hunt syndrome was favored. Under the latest course of steroids, the patient had noted some improvement in her facial pain; however, her vision in the right eye remained NPL.

When reviewing her imaging in a multidisciplinary orbital meeting, the bony erosion adjacent to the sphenoid sinus was outlined (Figures 1B, 1C), and a biopsy was recommended; however, it was considered to be too risky from both a neurosurgical and orbital approach. 

The patient was then referred to a rhinologist at a tertiary care center for advice regarding endonasal biopsy of the orbital apex lesion. Given the progressive deterioration in vision to NPL and ongoing pain, the biopsy benefit was deemed to outweigh potential risks of the procedure.

An image-guided right functional endoscopical sinus surgery (FESS), including antrostomy, sphenoethmoidectomy, and a transpterygoid approach to the inferior orbital fissure, was performed (six and a half months after her ED presentation). A left-sided vascularized nasoseptal flap was elevated and used to cover and remucosalize the dissected pterygoid fossa.

**Figure 3:**
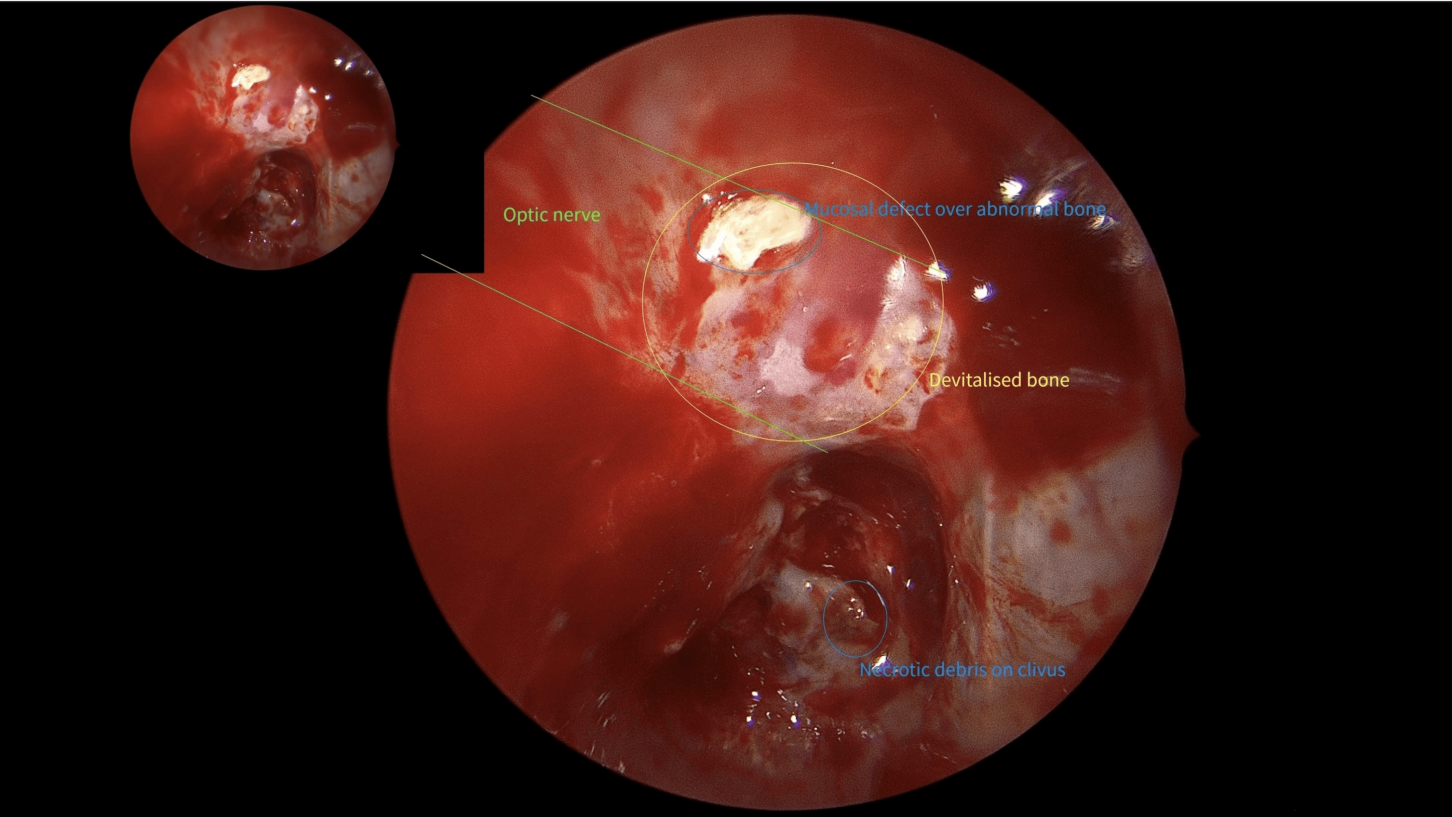
Functional endoscopical sinus surgery Intraoperative finding during functional endoscopical sinus surgery revealing patchy demineralized bone with overlying mucosal defects. Note the close relationship of the clivus to the optic nerve (ON) as a projection of the ON shows (green lines).

Intraoperatively, there were mucosal defects seen in the superolateral, posterior, and medial sphenoid sinus walls over abnormal appearing clival bone (Figure 3).

The StealthStation Navigation system (StealthStation®, Medtronic Sofamor Danek, Memphis, TN, USA) was utilized to identify the region of the inferior orbital fissure, which showed abnormal thickening and enhancement on MRI and was deemed to be the safest location for a diagnostic biopsy. Biopsies of the sphenoid sinus wall, clivus, and inferior orbital fissure were taken and sent for histopathology, microscopy, and culture.

**Figure 4:**
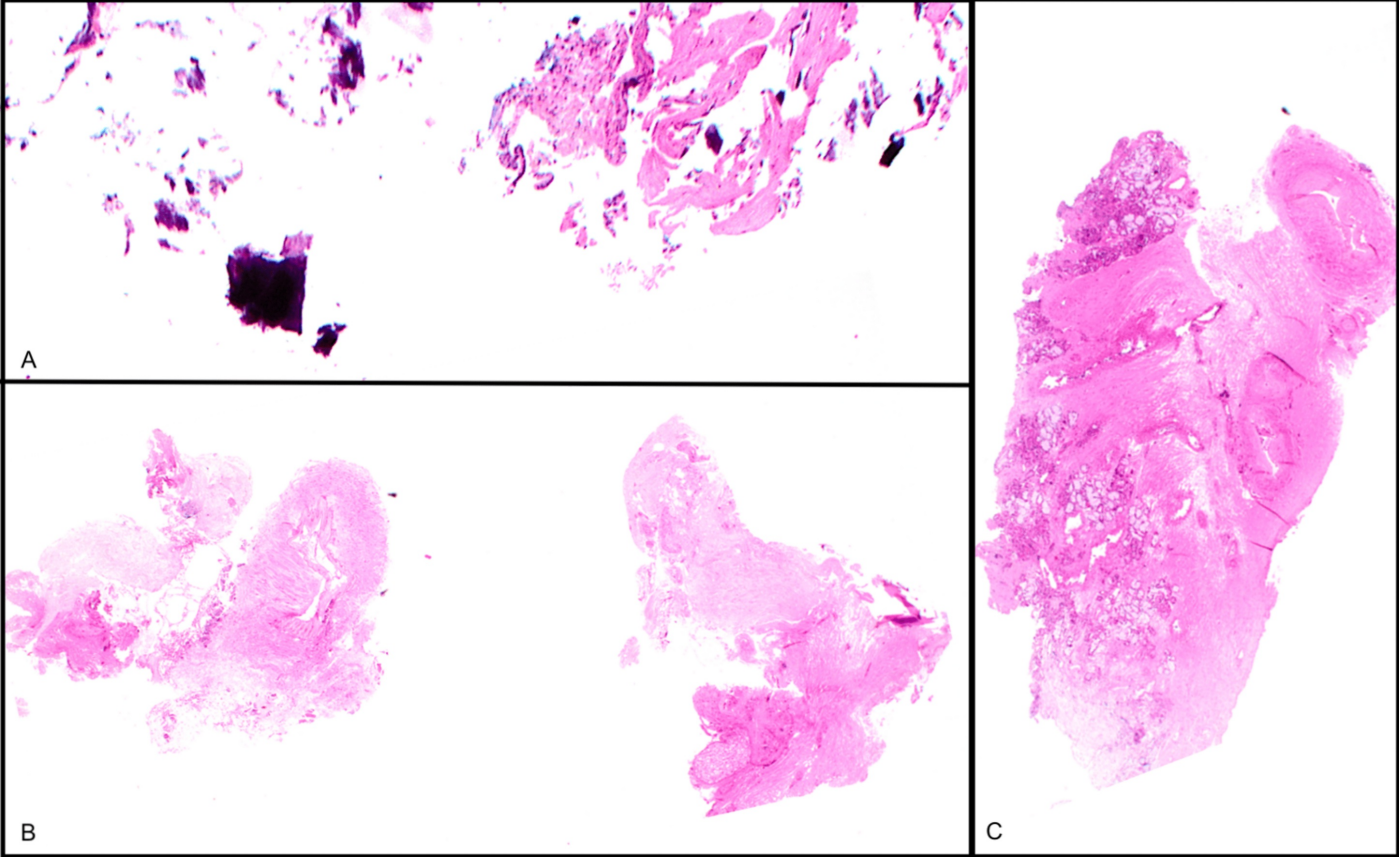
Biopsy samples A: clival bone; B: inferior orbital tissue; C: sphenopalatine artery (with surrounding stroma containing seromucinous glands), showing no inflammation or evidence of malignancy.

Direct microscopy of sinus samples taken from the clival bone, sphenopalatine artery, and inferior orbital tissue did not reveal inflammation or fungi on Grocott-Gomori methenamine silver (GMS) stain, and there was no evidence of malignancy (Figure 4). 

Microscopy and culture wound swab tissue specimens showed moderate growth of *Nocardia veterana* with typical filamentous morphology, including beading and branching, light growth of *Corynebacterium accolens*, and one colony of *Staphylococcus epidermidis*. A unifying diagnosis of nocardial clival osteomyelitis was made.

More than nine months after the onset of her first symptoms (and seven months after ED presentation), the patient received targeted therapy with imipenem and cilastatin intravenously and cotrimoxazole orally for six weeks, as recommended by infectious disease specialists, to be followed by oral antibiotics for six to 12 months. Her facial pain has subsequently resolved, but her ocular symptoms have persisted.

## Discussion

*Nocardia spp.* are filamentous, Gram-positive, aerobic bacteria that grow in water, decaying organic matter, and soil, and that may infect the pulmonary system or skin in immunocompromised patients. *Nocardia veterana* was first isolated in 2001 in an Australian veteran’s hospital (hence the name) and has been most frequently isolated in pulmonary infections. 

Clival osteomyelitis is a rare, life-threatening complication of untreated malignant otitis externa or paranasal sinus infection and is only rarely caused by* Nocardia spp*. 

To our knowledge, there are only four cases in the literature of sphenoid sinusitis caused by *Nocardia spp.*, with two of these reporting extension to the clivus [1-4]. However, these cases differ in many aspects from our patient (Table 1). 

**Table 1:** Case reports of Nocardia-induced sphenoid sinusitis Previous cases of *Nocardia*-induced sphenoid sinusitis, with or without skull base involvement. For clarity, treatment doses have been omitted and full titles of case reports listed under references [1-4]. TMP-SMX: trimethoprim-sulfamethoxazole; IV: intravenous; MRI: magnetic resonance imaging; CT: computed tomography

Publication	Pathogen	Infection Site and Key Findings	Immune Status	Age, Sex, and Pre-existing Conditions	Presenting Symptoms	Signs of Bony Erosion on Imaging (CT/MRI)	Summary of Therapy	Outcome
Roberts SA et al., Clin Infect Dis, 1995 [2]	Nocardia asteroides	Sphenoid sinusitis extending into the nasoethmoidal region, pituitary fossa, suprasellar cistern	Immunocompromised (azathioprine, prednisone)	35-year-old man; renal transplant for reflux nephropathy	1-year history of worsening occipital headaches, diplopia, lacrimation, nausea, left eye proptosis	Not mentioned (no new bony destruction post-treatment on CT)	Empirical oral sulfadimidine, bilateral sphenoidal sinusotomies; 8 weeks IV imipenem, 17 months oral erythromycin (later switched to roxithromycin)	Complete resolution of ophthalmoplegia
Giordano A et al., Eur Ann Otorhinolaryngol Head Neck Dis, 2016 [1]	Nocardia nova	Sphenoid sinusitis with infratemporal fossa abscess	Immunocompetent	72-year-old woman; well-controlled non-insulin-dependent diabetes, past breast adenocarcinoma	Severe left frontotemporal headache, left trigeminal neuralgia (after left maxillary dental implants), afebrile	Yes (CT, later during disease course)	Oral trimethoprim-sulfamethoxazole after empirical IV cefotaxime/clindamycin	Complete resolution
Abou-Al-Shaar H et al., Acta Neurochir (Wien), 2019 [3]	Nocardia abscessus, Nocardia exalbida, Nocardia gamkensis	Clival osteomyelitis secondary to sphenoid sinusitis	Immunocompetent	74-year-old woman; history of rheumatic fever	Headaches, abducens and hypoglossal nerve palsies, facial numbness, photophobia, neck stiffness	Yes (MRI)	Imipenem, linezolid, and trimethoprim-sulfamethoxazole (TMP-SMX); later tailored to ceftriaxone, TMP-SMX, and linezolid for 12 months	Complete recovery of diplopia, almost full recovery of tongue deviation
Villanueva DH et al., Cureus, 2023 [4]	Nocardia veterana	Clival osteomyelitis secondary to sphenoid sinusitis	Immunocompromised (tacrolimus, azathioprine, prednisone)	61-year-old woman; history of Sjögren’s disease, bilateral lung transplant for interstitial lung disease	Intractable headaches, right-sided facial tenderness refractory to analgesics and antibiotics	Not mentioned (MRI)	Empirical oral TMP-SMX, linezolid, IV cefepime; tailored to dual therapy with oral TMP-SMX and IV cefepime; 5 months later, TMP-SMX switched to oral minocycline; IV cefepime and oral minocycline completed at 12 months	Complete resolution

Nocardiosis typically occurs in immunocompromised patients. Only one of the other cases of nocardial clival osteomyelitis was in an immunocompetent patient [3], while a few other cases involving the sphenoid sinus [1] or other bones of the skull can be found in the literature [1,5-8]. Our patient was immunocompetent but had intermittently received corticosteroids during the course of the disease for a misdiagnosis of GCA. It is not possible to prove whether *Nocardia* was the cause of her initial symptoms or a secondary infection. However, isolated sphenoid sinus disease in general should raise the index of suspicion for atypical microbiology or underlying pathology, as up to 82% of the cases do not represent a common sinus bacterial pathogen [9]. *Nocardia* was therefore likely the initial cause of the sphenoid sinusitis despite her normal immunological status.

Isolated sphenoid sinusitis is known to constitute a risk factor for orbital symptoms due to its close anatomic relationship to the optic nerve, the cavernous sinus, and the orbit. A recent study has identified common risk factors for orbital involvement in isolated sphenoid sinusitis, which include diabetes mellitus, malignancies, or bony dehiscence on CT images [10]. Our patient developed chronic pain and then abducens nerve palsy after apparently adequate treatment for the sinusitis. 

Retrospectively, bone dehiscence was present before the onset of the abducens nerve palsy in our case. Unfortunately, its significance was not initially appreciated. It was noted when reviewing the patient’s imaging at an orbital multidisciplinary meeting, and as a result, the possibility of an atypical cause for sphenoid sinusitis with orbital involvement was raised.

To our knowledge, this seems to be the first case of *Nocardia veterana* sphenoid sinusitis leading to clival osteomyelitis and involvement of two cranial nerves. *Nocardia’s* affinity to the central nervous system (CNS) is well recognized, but this is more common after primary pulmonal infection. Severe facial pain and onset of CN palsies are typical symptoms for skull base osteomyelitis, with the abducens nerve being involved most frequently [11]. These symptoms, combined with bony destruction on imaging and a possibly prolonged history of symptoms, along with a lack of response to empirical therapy, should raise suspicion for atypical skull base osteomyelitis.

Diagnostics include CT, which may show nonspecific tissue swelling and bony erosion. The most consistent finding of MRI is clival hypo-intensity on T1-weighted images relative to normal fatty marrow [11]. Compared to other case reports of *Nocardia*-induced sphenoid sinusitis with or without accompanying osteomyelitis, similar imaging was used in this case. An earlier MRI scan might have detected the osteomyelitis, but unfortunately, the first MRI was not interpretable due to motion artifacts, and thus, these signs were either not evident early (on CT) or not at all (on MRI) in our case.

## Conclusions

Clival osteomyelitis should be included in the differential diagnosis of progressive symptoms (most frequently facial pain despite intense pain medication) in the setting of an underlying sphenoid sinusitis.

This case also demonstrates that awareness of a high likelihood of an atypical cause for isolated sphenoid sinusitis should be raised, and a low threshold for surgical intervention is justified, especially when bony erosions are evident on CT or when cranial nerve palsies occur. Early intervention with antibiotics with surgical sampling, and debridement is essential in the management of this rare entity to prevent further orbital or life-threatening complications.
